# A Lateral Flow Strip Based on a Truncated Aptamer-Complementary Strand for Detection of Type-B Aflatoxins in Nuts and Dried Figs

**DOI:** 10.3390/toxins12020136

**Published:** 2020-02-22

**Authors:** Zhilei Zhao, He Wang, Wenlei Zhai, Xiaoyuan Feng, Xia Fan, Ailiang Chen, Meng Wang

**Affiliations:** 1 Hebei University, No. 180 Wusi Dong Road, Lian Chi District, Baoding 071002, Hebei Province, China; zhaozl@hbu.edu.cn (Z.Z.); wanghe2262@163.com (H.W.); 2 Beijing Research Center for Agricultural Standards and Testing, No. 9 Middle Road of Shuguanghuayuan, Haidian Dist. Beijing 100097, China; zhaiwl@brcast.org.cn (W.Z.); fengxy@brcast.org.cn (X.F.); 3 Institute of Quality Standards and Testing Technology for Agro-products, Key Laboratory of Agro-product Quality and Safety, Chinese Academy of Agricultural Sciences, Beijing 100081, China; fanxia@caas.cn

**Keywords:** Type-B aflatoxins, lateral flow, aptamer, complementary strand, nuts and dried figs

## Abstract

Type-B aflatoxins (AFB_1_ and AFB_2_) frequently contaminate food, especially nuts and fried figs, and seriously threaten human health; hence, it is necessary for the newly rapid and sensitive detection methods to prevent the consumption of potentially contaminated food. Here, a lateral flow aptasensor for the detection of type-B aflatoxins was developed. It is based on the use of fluorescent dye Cy5 as a label for the aptamer, and on the competition between type-B aflatoxins and the complementary DNA of the aptamer. This is the first time that the complementary strand of the aptamer has been used as the test line (T-line) to detect type-B aflatoxins. In addition, the truncated aptamer was used to improve the affinity with type-B aflatoxins in our study. Therefore, the lengths of aptamer and cDNA probe were optimized as key parameters for higher sensitivity. In addition, binding buffer and organic solvent were investigated. The results showed that the best pair for achieving improved sensitivity and accuracy in detecting AFB_1_ was formed by a shorter aptamer (32 bases) coupled with the probe complementary to the AFB_1_ binding region of the aptamer. Under the optimal experimental conditions, the test strip showed an excellent linear relationship in the range from 0.2 to 20 ng/mL with a limit of detection of 0.16 ng/mL. This aptamer-based strip was successfully applied to the determination of type-B aflatoxins in spiked and commercial peanuts, almonds, and dried figs, and the recoveries of the spiked samples were from 93.3%−112.0%. The aptamer-complementary strand-based lateral flow test strip is a potential alternative tool for the rapid and sensitive detection of type-B aflatoxins in nuts and dried figs. It is of help for monitoring aflatoxins to avoid the consumption of unsafe food.

## 1. Introduction

Type-B aflatoxins, including aflatoxin B_1_ and B_2_ (AFB_1_ and AFB_2_), produced by *Aspergillus* species, have the highest toxicity among mycotoxins that seriously threaten the health of humans and animals [1]. AFB_1_ is highly carcinogenic, mutagenic, and teratogenic, as reported by the International Agency for Cancer (IARC) [2]. It is estimated that up to 28% of the total worldwide cases of hepatocellular carcinoma were caused by AFB_1_ [3]. In fact, consumption of nuts and dried fruits is one of the most important contributors to human exposure to mycotoxins, especially AFB_1_ [4]. According to data from the Rapid Alert System for Food and Feed (RASFF), there was a total of 460 border rejections in 2018 for mycotoxins, of which 90.2% were for aflatoxins, mainly from nuts, nut products, and figs. To ensure food safety and protect human health, many countries and regions in the world have set maximum allowable limits for AFB_1_ in nuts and dried figs, which are typically set as 2–8 μg/kg by the European Union [5], and 5–20 μg/kg by China [6]. However, a recent survey showed that the nuts and dried figs collected from China were seriously contaminated by AFB_1_, with the highest levels of 40.7 and 384.1 μg/kg [6], respectively, which far exceeded China’s limits. Therefore, it is necessary to develop rapid, sensitive, and reliable detection methods for type-B aflatoxins monitoring to prevent the consumption of insecure food.

Conventional approaches, including high-performance liquid chromatography (HPLC) [7,8] and HPLC-tandem mass spectrometry (HPLC-MS/MS) [5,6], have been widely used for AFB_1_ detection in nuts and dried fruits, but generally these methods need expensive instruments, highly skilled personnel, and complex pretreatment steps, making them difficult to use for real-time, fast and in-field detection. In addition to instrumental methods, immunoassays have proven to be effective in routine diagnostic applications for type-B aflatoxins. In particular, the lateral flow immunoassay (LFIA) is more attractive due to its advantages of visual observation, simple operations, and low testing costs [9]. However, classic LFIA detection uses antibodies as the bioreceptor elements, which are susceptible to physical and chemical conditions [10,11]. Aptamers are single-stranded DNA or RNA fragments obtained by the Systematic Evolution of Ligands by Exponential Enrichment (SELEX) [12]. Compared with antibodies, aptamers possess outstanding features, including non-immunogenicity, better thermal and chemical stability, high reproducibility, easy modification with various chemical groups, and a low synthesis cost [13,14,15]. Furthermore, aptamer-based lateral flow strips (ALFs) show more long-term preservation and cost effectiveness.

To date, AFLs have been reported for the detection of ochratoxin A [16,17,18], zearalenone [19] and AFB_1_ [20]. However, the existing AFLs was based on the competition between free AFB_1_ and immobilized hapten-albumin from bovine serum (BSA) for binding to the AFB_1_ aptamer. It is well known that the preparation for hapten conjugate is expensive, toxic, and difficult to synthesize. In addition, the truncated aptamer for AFB_1_ detection showed better performance than the parent aptamers [21]. Therefore, the aim of our study was to find suitable complementary strands to achieve a balance between reduced affinity to aptamer and high efficiency of hybridization with truncated aptamer, and then develop a fluorescent lateral flow strip based on a truncated aptamer-complementary strand for rapid and sensitive detection of type-B aflatoxins in nuts and dried figs. After optimization, this protocol was evaluated and validated by LC-MS/MS. To the best of our knowledge, this is the first time that the complementary strand of the aptamer was used as the test line (T-line) to detect type-B aflatoxins. 

## 2. Results and Discussion

### 2.1. Principles for the Aptamer-Complementary Strand-Based Lateral Flow Test Strip

**Figure 1:**
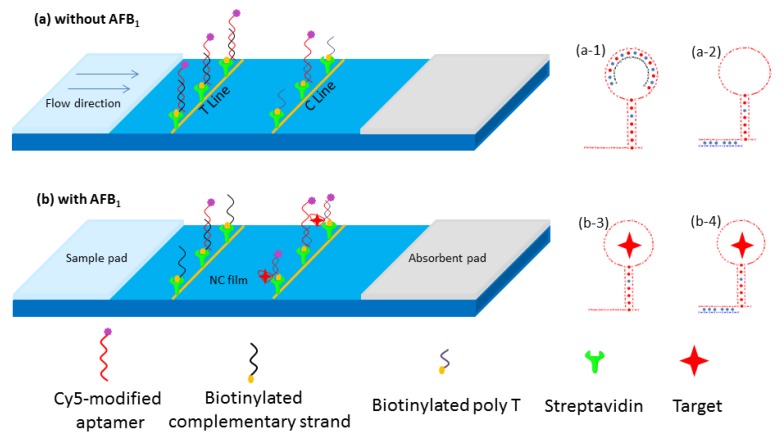
Schematic illustration of adapter side-flow tomography sensor for detecting aflatoxin B_1_ (AFB_1_). (**a**) In the absence of AFB_1_, the Cy5-labeled aptamer was first combined with a partially complementary strand at the test-line (T-line) (**a-1**); the remaining aptamer was then combined with the poly T of the control line (C-line) (**a-2**). (**b**) In the presence of AFB_1_, the Cy5-labeled aptamer preferred to bind with AFB_1_ (**b-3**), and the fluorescence intensity of the T-line decreased. The aptamer bound to AFB_1_ was still bound to poly T of the C-line (**b-4**), while the fluorescence intensity of the C-line increased, and the value of test-line to control line (T/C) was used to quantify AFB_1_.

**Figure 2:**
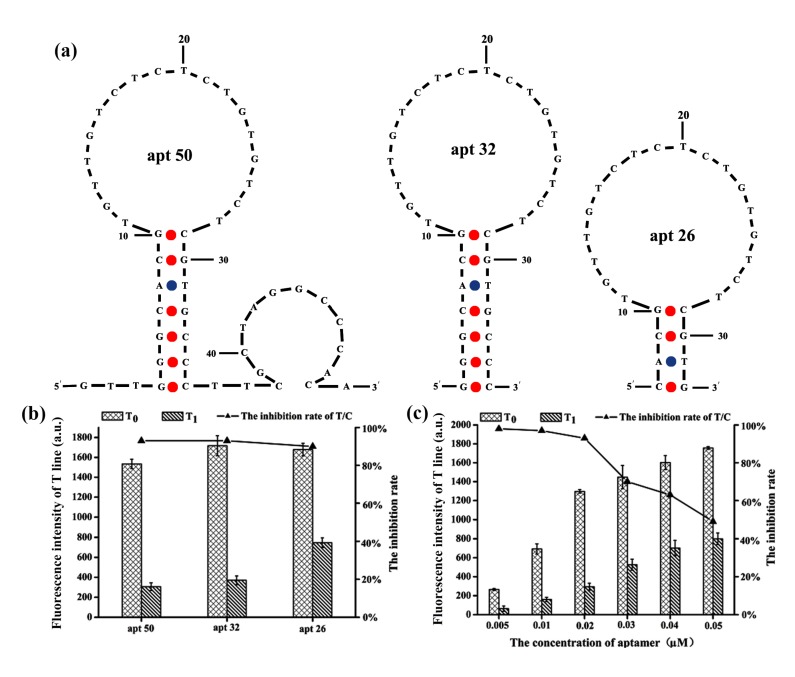
Optimization of aptamer sequences and concentrations (T_0_ and T_1_ correspond to the fluorescence intensity of T-line in the absence and presence of 100 ng/mL AFB_1_). (**a**) The predicted secondary structure of the 50-mer aptamer (apt 50), apt 32, and apt 26. The apt 32 and apt 26 probes were truncated from the apt 50 [21]. (**b**) Fluorescence intensity of T-line and the inhibition rates of apt 50, apt 32, and apt 26 with and without AFB_1_. (**c**) Fluorescence intensity of T-line and the inhibition rates with different concentrations of apt 32.

**Figure 3:**
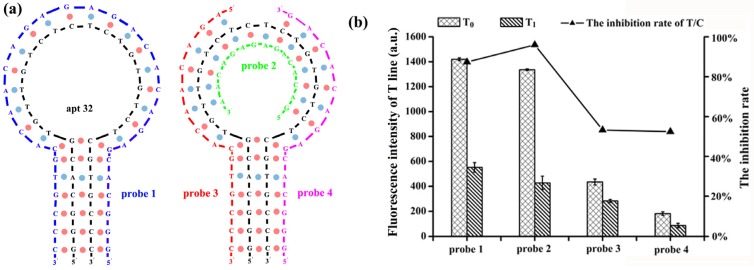
Optimization of the complementary sequence on the T-line (T_0_ and T_1_ correspond to the fluorescence intensity of T-line in the absence and presence of 100 ng/mL AFB_1_). (**a**) The complementary relationship of probe 1, probe 2, probe 3, and probe 4 with apt 32. (**b**) The variation of the fluorescence intensity of T-line and inhibition rate with different complementary DNA probes.

The principle for the aptamer-based lateral flow test strip (ALFs) was based on the competition for Cy5-Apt between the complementary strand on the test-line and AFB_1_ in the samples (Figure 1). In the absence of AFB_1_, the solution was chromatographed from the sample pad to the nitrocellulose (NC) membrane, and the Cy5-labeled aptamer was first combined with a partially complementary strand at the test-line, as shown in Figure 1a-1. The remaining aptamer was then combined with the poly T (Table 1) of the control line, as shown in Figure 1a-2, which resulted in a higher fluorescence value of the test-line and a lower fluorescence value of the control line. Conversely, in the presence of AFB_1_, the Cy5-labeled aptamer preferred to bind with AFB_1_ (Figure 1b-3), rather than couple with the partially complementary strand at the test-line (Figure 1b). In other words, the more aptamer-AFB_1_ complexes there were in the solution, the less free Cy5-labeled aptamer was captured on the test-line. As a result, the fluorescence intensity of the test-line was decreased. Moreover, aptamer probes would hybridize with poly T on the control line in any case, ensuring the detection validity. Quantitative analysis was performed by measuring the fluorescent intensity of the two lines with analysis software, and the intensity ratio of the test-line to control line (T/C) was inversely proportional to the AFB_1_ concentration in the samples.

### 2.2. Optimization of the Aptamer Length and Concentration

The length of AFB_1_ aptamer is a key factor for consideration when rationally designing a structure-switching aptamer [22,23]. A previous study showed that the short segment close to the 3’ end of the 50-mer AFB_1_ aptamer was not essential for target binding [21], and the use of the shorter aptamer could save costs without compromising the AFB_1_ recognition capability. Based on this result, the length of AFB_1_ aptamer was optimized with ALFs analysis. The DNA sequences of the aptamers are presented in Table 1. The apt 32 and apt 26 for AFB_1_ binding in our study were truncated from the reference aptamer (apt 50), which was first reported by Le et al. [24], and their predicted secondary structures are shown in Figure 2a. The three Cy5-labeled aptamers were mixed with the AFB_1_ standard of 100 ng/mL at a ratio of 1:9. Fluorescence intensities were measured with and without the target. As shown in Figure 2b, there was no significant difference in the T/C values among three aptamers. However, for the T-line, the fluorescence intensity of apt 50 and apt 32 added to the target was lower than that of apt 26, indicating weaker binding between AFB_1_ and apt 26. All three aptamers could bind with the AFB_1_, which further confirmed that the sequence of the loop region is important for aptamer binding affinity. Moreover, compared with apt 26, apt 32 contains three more G-C base pairs, which may further stabilize the secondary structure and strengthen the binding towards AFB_1_. The previous study also showed that the proper numbers of base pairs in the stem region is required for the aptamer to have strong binding affinity to AFB_1_, which is helpful to form a stable stem loop structure [21]. Judging by the performance of these three aptamers, apt 32 was selected as the AFB_1_ binding aptamer in this study.

The concentration of the aptamer has a great influence on the sensitivity and detection limit of the test strip. In order to determine the optimal aptamer concentration, the target was mixed with different concentrations of apt 32 (0.005, 0.01, 0.02, 0.03, 0.04, 0.05 μM) in a ratio of 9:1, and 60 μL mixture was loaded onto the test strip. The T/C inhibition rate was used to evaluate the analytical performance. The fluorescence intensities (T and C-line) were gradually enhanced with the increase of aptamer levels, but the T/C inhibition rate sharply decreased from 0.02 to 0.05 μM apt 32 (Figure 2c). Moreover, there was no significant difference in the T/C inhibition rate from 0.005 to 0.02 μM apt 32. Since the T-line fluorescence intensity with 0.005 and 0.01 μM of aptamer concentration was low, this may lead to large systematic errors. Hence, 0.02 μM apt 32 was the best choice for the optimal concentration for further studies.

### 2.3. Optimization of the Complementary Strand on the T-Line

Apart from forming a complex with its target, the aptamer can also hybridize with a complementary DNA strand. Therefore, the sequence of the complementary strand could also have a significant impact on the interaction between aptamer and target [22,23]. Ideally, the complementary strand on the T-line should be able to capture free aptamer in the absence of target. On the other hand, it should not compete with the target for aptamer binding. In this study, we synthesized four types of complementary DNA modified with biotin. The sequences of the complementary DNA are listed in Table 1, which include one full complement sequence (probe 1) with apt 32, and three partial complementary sequences (probe 2, probe 3, and probe 4) as DNA probes. The length of each partial complementary (Figure 3a) strand was 15 bases. All DNA probes were tested to identify the optimal DNA probe as the complementary strand for the ALFs assay. As shown in Figure 3b, in the presence of 100 ng/mL AFB_1_ standard, the T-line fluorescence intensity of all four DNA probes decreased. However, the inhibition rates of probe 3 and 4 were significantly lower than that of probe 1 and 2, suggesting that neither of them is suitable for serving as the complementary strand. In the case of probe 1 and 2, the inhibition rates were calculated as 87.4% (probe 1) and 95.9% (probe 2), respectively. The relatively lower inhibition rate of probe 1 might be caused by the competitive binding between the complementary strand and AFB_1_ target. A previous study showed that the aptamer tends to hybridize more strongly with complementary DNA than a target if the length of complementary DNA is the same as that of an aptamer [25]. Based on this result, probe 2, with the highest inhibition rate, was chosen as the complementary strand for the T-line. 

### 2.4. Optimization of Reaction Conditions between Aptamer and AFB_1_

The composition of the binding buffer has a crucial effect on spatial conformation of the aptamer, which directly affects the binding strength between aptamer and target. Therefore, the composition of the binding buffer was also optimized in this study. Firstly, three commonly used binding buffers (Tris-HCl, HEPES, and PBS) were compared, then the concentration of Mg^2+^ was investigated. Finally, the type and maximum concentration of organic solvent in the binding buffer was evaluated.

**Figure 4:**
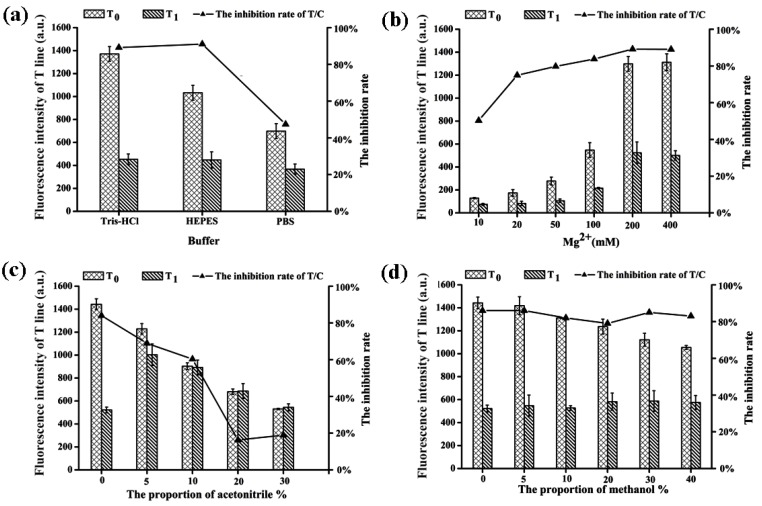
Optimization of the type and composition of the binding buffer (T_0_ and T_1_ correspond to the fluorescence intensity of T-line in the absence and presence of 100 ng/mL AFB_1_). (**a**) The trend of fluorescence intensity and inhibition rates with three different binding buffers. (**b**) The trend of fluorescence intensity and inhibition rates with different concentrations of Mg^2+^ in Tris-HCl buffer. (**c**) The variation of fluorescence intensity and inhibition rates with the increase of acetonitrile concentration. (**d**) The variation of fluorescence intensity and inhibition rates with the increase of methanol concentration.

The ideal buffer can improve the specificity and stability of the test strips, and thus the fluorescence intensity of C-line was measured in order to determine the binding strength between the aptamer and poly-T in different buffer systems. Among the three tested buffers, Tris-HCl and HEPES provided similar inhibition rates (89.2% and 91.1%, respectively), while the inhibition rate was significantly lower in the PBS buffer system (Figure 4a). On the other hand, the T-line fluorescence intensity of Tris-HCl buffer was higher than that of HEPES buffer. According to this result, Tris-HCl buffer was identified as the binding buffer for the subsequent experiments. 

The concentration of Mg^2+^ has a significant effect on the hybridization of DNA strands, it also affects the binding affinity of targets and aptamers by influencing DNA structure [26,27]. For the purpose of optimizing Mg^2+^ concentration in our system, binding buffers with different concentrations of MgCl_2_ (10, 20, 50, 100, 200, 400 mM) were prepared. As the concentration of Mg^2+^ increased, not only did the fluorescence intensity of T-line increase significantly, but also the inhibition rate increased from 50.3% to 89.1% (Figure 4b). As a result, 200 mM of Mg^2+^ in Tris-HCl buffer was identified to be the optimal concentration.

Acetonitrile and methanol are widely introduced to address the poor solubility of AFB_1_ in aqueous medium. However, an excess amount of organic solvent might lead to incomplete binding between DNA strands [28] and could even brake down the structure of the NC membrane. Therefore, it is necessary to determine which type of organic solvent is more suitable for this sensor. Moreover, the maximum ratio of organic solvent should also be determined. In this experiment, different volumes of acetonitrile or methanol were added to prepare the binding buffer with 0, 5, 10, 20, 30, and 40% of organic solvent, respectively. The results in Figure 4c showed that the addition of acetonitrile greatly affected the binding between the aptamer and target, as the inhibition rate obviously decreased from 84.0% to 16.3% with the increase of acetonitrile concentration (0 to 20%). In contrast, the inhibition rate was basically unchanged upon addition of methanol (Figure 4d). Further comparing of T-line fluorescence intensity revealed that the concentration of methanol should not exceed 10% to guarantee the minimum interference induced by the solvent.

### 2.5. Specificity Test

**Figure 5:**
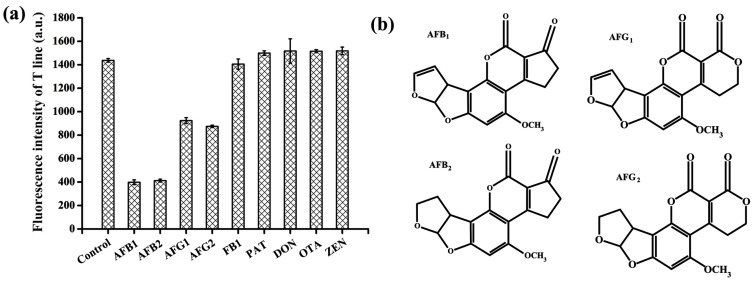
Specificity of the aptamer-based lateral flow strips (ALFs) by analyzing AFB_1_ against other mycotoxins at 100 ng/mL. (**a**) The T-line fluorescence intensity of other mycotoxins. (**b**) Chemical structures of AFB_1_, AFB_2_, aflatoxin G_1_ (AFG_1_) and AFG_2_.

The specificity towards the target AFB_1_ by using our developed ALFs was studied. Several relevant mycotoxins including AFB_2_, aflatoxin G_1_ (AFG_1_), AFG_2_, ochratoxin A (OTA), zearalenone (ZEN), fumonisin B_1_ (FB_1_), and deoxynivalenol (DON) were measured at the same concentration of 100 ng/mL. As shown in Figure 5a, the aptamer showed similar high binding affinity to AFB_1_ and AFB_2_. No change of T-line fluorescence intensity was observed compared with the control for the other types of mycotoxins, which indicated that the ALFs had high specificity for type-B aflatoxins. Furthermore, it showed much decreased binding strength to AFG_1_ and AFG_2_. The T-line fluorescence intensity of AFG_1_ and AFG_2_ was 2.3- and 2.2-fold higher than that of AFB_1_, respectively. The distinction of AFB_1_ and AFB_2_ proved to be challenging because of the high similarity between their chemical structures (Figure 5b). Presumably, the C-C double bond on one of the furan rings of AFB_1_ does not participate in the aptamer binding. However, it is clear that the tested sensor showed high binding affinity to these two groups of aflatoxins than other types of mycotoxins. More importantly, the ALFs can simultaneously detect both AFB_1_ and AFB_2_, which is an advantage for food security.

### 2.6. Quantitative Analysis of Type-B Aflatoxins

**Figure 6:**
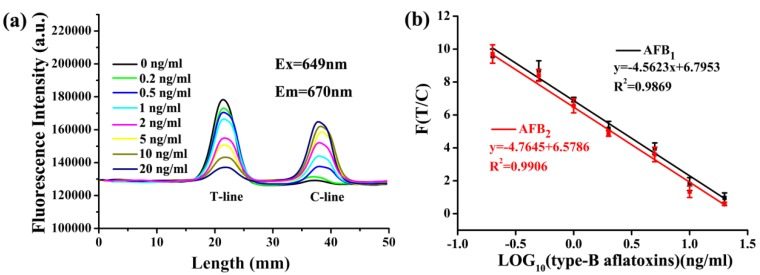
Quantitative determination of type-B aflatoxins with prepared strips. (**a**) Fluorescence spectra of T-line and C-line at different concentrations of AFB_1_. (**b**) The linear relationship between the ratio of fluorescence intensity (F(T/C)) of the detection line and the control line and the logarithm of the concentration of type-B aflatoxins at different concentrations.

Under the optimized conditions, the sensitivity of the test strip was examined. The AFB_1_ and AFB_2_ stock solution were diluted into the concentrations of 0.2, 0.5, 1, 2, 5, 10, 20 ng/mL. The standard solutions were mixed with 0.02 μM aptamer solution with the ratio of 9:1. After 10 min, the mixed solution was loaded onto the test strip and subsequently scanned with a card reader. It was shown that, as the concentration of type-B aflatoxins increased, the fluorescence intensity of the T-line gradually decreased, while that the fluorescence intensity of the C-line increased, which resulted in the value of T/C decreasing (Figure 6a). The calculated F (T/C) value had a good linear relationship with the logarithm of the type-B aflatoxins concentration. A linear range between 0.2 and 20 ng/mL was determined for AFB_1_ and AFB_2_ with a correlation coefficient (*r*) of 0.9869 and 0.9906, respectively (Figure 6b). The limit of detection (LOD) was determined to be as low as 0.16 ng/mL. This result demonstrated that our test strip is capable of quantitative analysis of type-B aflatoxins at an extremely low level. Although the sensitivity of our developed ALFs is similar to that of a previous study (Table 2), this is the first time that ALFs have been applied for the simultaneous detection of AFB_1_ and AFB_2_. Moreover, the other benefit of these ALFs is that the complementary strand instead of hapten-BSA as test line can save costs, and facilitate synthesis and reproducibility.

### 2.7. Analysis of Type-B Aflatoxins in Foods 

To evaluate the reliability and accuracy of the prepared ALFs biosensors, peanuts, almonds, and dried figs samples were spiked with type-B aflatoxins standard solution at a concentration of 3 and 10 μg/kg. The spiked samples were processed by simply extracting with acetonitrile and filtering through an solid phase extraction (SPE) cartridge. The extracts were re-dissolved in binding buffer and analyzed by our ALFs. The recovery rates were summarized in Table 3. The recovery range of the different matrix for AFB_1_ was 96.5%–109.4% with the relative standard deviation (RSD) of 2.0% to 6.5%; for AFB_2_ it was 93.3%–112.0%, and the RSD ranged from 2.7% to 7.9%. The limit of quantitation (LOQ) was determined to be 0.5 μg/kg, which is far below the European Union (EU) limit. This result matches well with the standard HPLC-MS/MS method. It is proven in this study that the proposed ALFs are capable of detecting type-B aflatoxins in food samples. Seventeen samples of peanuts, dried figs, and almonds were selected and extracted. The extracts were measured using ALFs and HPLC-MS/MS, respectively. The comparison results showed that AFB_1_ and AFB_2_ were not detected from the collected samples, and the results of the two detection methods were consistent. 

## 3. Conclusions

In summary, we introduced an ultrafast, sensitive, specific, and easy-to-operate method for type-B aflatoxins analysis by designing and preparing a lateral flow strip biosensor based on the specific recognition of type-B aflatoxins by an aptamer labeled with a fluorescent tag. This sensor improved the sensitivity and accuracy towards AFB_1_ by employing a shortened aptamer sequence, which made it more suitable for practical applications. The fluorescence intensity on the strip could be recorded by a portable strip reader. For quantitative detection, the ratio of fluorescence intensities between T and C-line provided a good linear relationship with the logarithm of the type-B aflatoxins concentration (0.2–20 ng/mL). By using this ALFs sensor, rapid detection of type-B aflatoxins could be achieved within 20 minutes. Finally, peanuts, almonds, and dried figs samples were spiked with type-B aflatoxins and analyzed using this method. The result verified that our ALFs could satisfy the requirement of on-site detection of spiked samples. Compared with the traditional HPLC-MS/MS technique, the reported sensing device has the advantages of being low cost, fast to analyze and easy to operate, which make it more suitable for on-site and rapid monitoring of type-B aflatoxins contamination in food samples. Moreover, this strategy is also applicable to construct test strips for other mycotoxins.

## 4. Materials and Methods 

### 4.1. Reagents and Instruments

Aflatoxin B_1_ (AFB_1_), aflatoxin B_2_ (AFB_2_), aflatoxin G_1_ (AFG_1_), aflatoxin G_2_ (AFG_2_), ochratoxin A (OTA), zearalenone (ZEN), fumonisin B_1_ (FB_1_), and deoxynivalenol (DON) were purchased from Romer international trading Co., Ltd (Beijing, China). Streptavidin was ordered from Solarbio Biological Technology Company (Beijing, China). PEG 20000, Tween-20 and sucrose were bought from J&K Scientific Ltd (Beijing, China). Tris (hydroxymethyl) aminomethane and 4-hydroxyethylpiperazineethanesulfonic acid (HEPES) were purchased from Sigma-Aldrich (Beijing, China). All other chemicals were purchased from Beijing Chemical Reagent Company (Beijing, China). All reagents were of analytical grade and used without further purification or treatment unless specified.

The composition of binding buffer A was 10 mM Tris, 120 mM NaCl, 5 mM KCl, and 200 mM MgCl_2_. The composition of binding buffer B was 10 mM HEPES and 200 mM MgCl_2_. Binding buffer C was made up of 8 mM Na_2_HPO_4_, 136 mM NaCl, 2 mM KH_2_PO_4_, 2.6 mM KCl, and 200 mM MgCl_2_. All three buffers contained 5% methanol (*v/v*), 1% PEG 20000 (*m/v*), 2% sucrose (*m/v*), 0.1% Tween-20 (*v/v*), and the pH was adjusted to 7.4.

The absorbent pad (H5076), sample pad (GL-b04), nitrocellulose (NC) membrane (Sartorius CN140), and PVC plastic adhesive backing used in the experiments were purchased from Shanghai Jieyi Biotechnology Co., Ltd, China. Each lateral flow chromatography strip was cut using a 3050 dispensing platform and KM-3100 Cutter (BioDot, Irvine, CA, USA). The fluorescence signals of the test strip were scanned by an ESEQuant LR3 FL Premium reader (Qiagen, Frankfurt, Germany).

The AFB_1_ aptamer sequence [23,24] and complementary probe sequences used in this project were listed in Table 1. All DNA probes were synthesized by Sangon Biotechnology Co., Ltd. (Shanghai, China).

### 4.2. Preparation of ALFs

The ALFs are composed of four parts: a sample pad, a NC membrane, an absorbent pad, and a PVC plastic adhesive backing. The sample pad, NC membrane, and absorbent pad is sequentially attached to the PVC plastic adhesive backing from left to right according to the direction of migration. The sample pad and absorbent pad sequentially laminate 3 mm with the NC membrane to guarantee smooth migration of solutions. The NC membrane was used to prepare test zones.

The NC membrane can only immobilize proteins on its surface, so the detection probe and the control probe labeled with biotin are reacted with streptavidin to form a biotin-streptavidin conjugate, which was anchored on the NC membrane in advance. The 3 μM biotin labeled partial complementary strand (biotin-probe 1) and 3 μM poly-T (biotin-probe 2) were mixed separately with streptavidin (1 mg mL^−1^) by 7: 1 (*v/v*) and incubated for 2 hours at room temperature. The streptavidin-biotin labeled DNA probe 1 (SA-biotin probe 1) and probe 2 (SA-biotin probe 2) were dispensed on the NC membrane of test (T) line and control (C) line using the 3050-dispensing platform, respectively. The distance between the two lines is 5 mm. It was dried at 37 °C for 1 h and the whole assembled sheet was cut into 4 mm strips by KM-3100 Cutter, and then stored in a desiccator before use.

### 4.3. Type-B Aflatoxins Analysis

The sample was mixed with Cy5-labeled aptamer in an Eppendorf tube at a ratio of 9:1 (*v/v*) and incubated at room temperature for 10 min. After that, each 60 µL portion of the mixture was loaded on the sample pad of an ALFs. All liquid was allowed to migrate freely along the strip until absorbed by the absorbent pad. After 10 min, the fluorescence intensity on the T and C-lines was recorded by scanning the test strip with ESEQuant LR3 FL Premium Reader.

### 4.4. Optimization of Test Conditions

In order to achieve the desirable performance of the ALFs, a series of impact factors that potentially affect the T and C-lines in the test strip were optimized, including the lengths and concentrations of the aptamer, the sequence of the complementary strands and the compositions of binding buffer solution (including which type of buffer, the concentration of Mg^2+^, and the ratio of organic solvent). The inhibition rate was introduced for evaluating the performance of the sensors under different conditions. It was calculated following the formula:

(1) $$inhibition\;rate=\left(\frac{T_{0}}{C_{0}}-\frac{T_{1}}{C_{1}}\right)/\frac{T_{0}}{C_{0}}$$

T_0_ and C_0_ correspond to the fluorescence intensity of T and C-line in the absence of AFB_1_, while T_1_ and C_1_ represent the fluorescence intensity of T and C-line in the presence of 100 ng/mL AFB_1_. In the case of conditions with similar inhibition rates, the value of T_1_ was compared to determine the optimal condition.

For quantitative analysis, the ratio of T and C fluorescence intensities was calculated as the concentration evaluation index of type-B aflatoxins, which was used to draw the standard curve.

### 4.5. Analysis of Food Samples

In this study, peanut, almond, and dried figs were chosen as food samples to further confirm the outcome of practical application by spiked recovery. A total of 17 samples consisting of peanuts (9), almonds (3), and dried figs (5) were randomly collected from different supermarkets and local markets in Beijing, China. The extraction of the specimen was performed following previous work [32]. In detail, 5.0 g of sample spiked with known amount of AFB_1_ was placed into a 50 mL polypropylene centrifugation tube and diluted with 25 mL 80% acetonitrile aqueous solution containing 100 mM citric acid. After that, the tube was transferred to a shaker and shaken at room temperature with a speed of 150 rpm for 30 minutes. Subsequently, the sample was centrifuged at 10,000 rpm for 10 min at 4 °C using a centrifuge. A volume of 5.0 mL supernatant layer was aspirated and filtered through a SPE cartridge column. Finally, the collected solution was evaporated to dryness at 50 °C under nitrogen atmosphere, and re-dissolved in 1 mL binding buffer as the extract solution. For each measurement, a mixture containing 54 µL of the extract solution and 6 µL of the Cy5 labeled aptamer solution was added on the aptamer-based flow chromatography test strip. The average recovery was calculated by comparing with the actual concentration of type-B aflatoxins.

## Figures and Tables

**Table 1:** Sequences of oligonucleotides used in the study.

Name	Sequence (5’–3’)
apt 50	Cy5-A_12_-GTTGGGCACGTGTTGTCTCTCTGTGTCTCGTGCCCTTCGCTAGGCCCACA
apt 32	Cy5-A_12_-GGGCACGTGTTGTCTCTCTGTGTCTCGTGCCC
apt 26	Cy5-A_12_-CACGTGTTGTCTCTCTGTGTCTCGTG
probe 1	biotin-GGGCACGAGACACAGAGAGACAACACGTGCCC
probe 2	biotin-GACACAGAGAGACAA
probe 3	biotin-GGGCACGAGACACAG
probe 4	biotin-AGACAACACGTGCCC
poly T	biotin-TTTTTTTTTTTT

**Table 2:** Summary of the analytical performance of different test strip detection methods for AFB_1_ based on the AFB_1_−BSA as the Test Line.

Method	Test Line	Range (μg/kg)	LOD^1^ (μg/kg)	Sample	Reference
Antibody-strip	AFB_1_−BSA	0.3–10	0.1	Soybean sauce	[29]
Antibody-strip	AFB_1_−BSA	0.3–1	0.3	Agricultural products	[30]
Antibody-strip	AFB_1_−BSA	–	0.1	medicinal materials	[31]
Aptamer-strip	AFB_1_−BSA	0.1–1000	0.1	food and feedstuffs	[20]
Aptamer-strip	DNA single strand	0.2–20	0.16	nuts and dried figs	present

^1^LOD: limit of detection

**Table 3:** Detection results of the type-B aflatoxins levels in spiked peanut and fig samples.

Sample	Mycotoxins	Spiked (μg/kg)	HPLC-MS/MS Detected (μg/kg)	RSD^1^ (%)	Proposed Method Detected (μg/kg)	Recovery (%)	RSD (%)
Peanut	AFB_1_	3	2.9	4.1	3.1	103.7	2.2
		10	10.1	2.9	9.6	96.5	6.5
	AFB_2_	3	3.1	1.4	3.0	101.0	3.3
		10	10.5	0.8	10.9	108.7	6.9
Dried figs	AFB_1_	3	2.9	3.6	3.3	109.4	2.0
		10	9.5	3.2	10.2	102.2	2.2
	AFB_2_	3	3.1	0.47	2.8	93.3	5.9
		10	10.5	0.57	11.4	112.0	2.7
Almond	AFB_1_	3	2.9	0.4	3.1	102.2	2.2
		10	10.0	2.0	9.8	97.6	4.9
	AFB_2_	3	2.7	1.2	2.8	93.7	4.1
		10	9.89	0.6	9.2	93.5	7.9

^1^ RSD: relative standard deviation.
